# Valproate hampers podocyte acquisition of immune phenotypes via intercepting the GSK3β facilitated NFkB activation

**DOI:** 10.18632/oncotarget.19917

**Published:** 2017-08-03

**Authors:** Pei Wang, Sijie Zhou, Yan Ge, Minglei Lu, Zhangsuo Liu, Rujun Gong

**Affiliations:** ^1^ Institute of Nephrology, Blood Purification Center, the First Affiliated Hospital of Zhengzhou University, Zhengzhou, China; ^2^ Department of Medicine, Division of Kidney Disease and Hypertension, Brown University School of Medicine, Providence, Rhode Island, USA

**Keywords:** glomerulus, podocyte, immune phenotype, proteinuria, NFkB, Pathology Section

## Abstract

Glomerular podocytes are able to transdifferentiate under disease conditions, acquire *de novo* immune phenotypes and behave as immunocompetent cells, like phagocytes or antigen-presenting cells. Upon stimulation with lipopolysaccharide (LPS), a prototypical pathogen-associated molecular pattern, podocytes demonstrated *de novo* expression of a variety of NFkB-dependent immune molecules that are pivotal for immune response, including major histocompatibility complex (MHC) class II, costimulatory molecule CD80, lysosomal protease cathepsin L as well as CC chemokine ligand 2 and 5, ultimately resulting in podocyte dysfunction, characterized by cellular shrinkage, a spindle-like or asterlike cell shape and impairment of actin cytoskeleton integrity. The LPS-elicited podocyte phenotypic changes were concurrent with nuclear factor (NF) kB phosphorylation, which was associated with glycogen synthase kinase (GSK) 3β overactivity, marked by a diminished inhibitory phosphorylation of GSK3β. In contrast, valproate, an anticonvulsant and mood stabilizer, offset GSK3β overactivity in LPS-injured podocytes and mitigated NFkB activation and podocyte acquisition of immune phenotypes as well as the ensuing cytopathic changes, podocyte cytoskeleton disorganization and dysfunction. The protective effect of valproate was strikingly blunted in podocytes expressing the constitutively active GSK3β, suggesting an essential role of inhibitory phosphorylation of GSK3β. In vivo in LPS-injured mice, valproate therapy abolished GSK3β overactivity in glomeruli and attenuated podocyte injury and albuminuria, concomitant with a lessened NFkB activation and diminished induction of diverse podocytopathic immune molecules in podocytes and glomeruli. Taken together, valproate directly protects against podocyte injury and hampers podocyte acquisition of *de novo* immune phenotypes *via* intercepting the GSK3β facilitated NFkB activation.

## INTRODUCTION

As a key structural component of the glomerular filtration barrier, glomerular podocytes play a critical role in maintaining the homeostasis of glomerular filtration rate and glomerular permselectivity [1-3]. Podocyte dysfunction or injury could be induced in a variety of acquired or congenital diseases by diverse immune or non-immune mediated mechanisms, such as podocytopathic effects of permeability factors generated following cellular or humoral immune reactions [4], direct podocyte toxicity caused by nephrotoxic substances [3], hemodynamics-elicited mechanical stress, oxidative damage, metabolic stress and others. Recently, a growing body of evidence suggests that podocytes, upon injury, may transdifferentiate and acquire *de novo* immune cell-like phenotypes [5-14]. By far, a number of immune molecules have been reported to be newly expressed in podocytes under diverse disease conditions, such as systemic autoimmune disease like lupus [9], primary glomerulopathies like membranous nephropathy or focal segmental glomerulosclerosis (FSGS) [15], or metabolic diseases like diabetes [16]. The *de novo* immune molecules consist of diverse proinflammatory cytokines, such as interleukin-1 a and β [5], and chemokines, like CC chemokine ligands (CCL) 2 and 5 [6, 7]. Besides, multiple immune cell surface marker proteins that are pivotal for mediating cell-mediated immune response, such as major histocompatibility complex (MHC) class II [8], dendritic cell-specific intercellular adhesion molecule-3-grabbing non-integrin [9], and the costimulatory molecule CD80 [11], have also been shown in diseased podocytes. Moreover, lysosomal protease cathepsin L [12], which is primarily found in leukocytes and crucial for immune reactions [17], could be induced in stressed podocytes. The exact role of these newly acquired immune phenotypes in the pathogenesis of podocyte or glomerular injury remains unclear. But there is accumulating evidence suggesting that podocytes may behave as nonhematopoietic professional antigen-presenting cells that are able to trigger adaptive immune responses [10, 13-15]. On the other hand, many immune molecules, like the costimulatory molecule CD80 and lysosomal protease cathepsin L, function at the interface between innate/adaptive immunity and cytoskeletal remodeling [11, 18]. *De novo* expression of these immune molecules has been associated with impairment of cytoskeletal integrity in podocytes [11, 12, 18, 19], denoting that podocyte acquisition of these immune phenotypes may be pathogenic. Of note, nuclear factor (NF) kB signaling is commonly essential for expression of all immune molecules [20]. The NFkB-driven expression of numerous NFkB-dependent immune molecules is finely regulated by glycogen synthase kinase (GSK)3β [21, 22], which is a well-conserved, ubiquitous serine/threonine protein kinase originally characterized as one that regulates glucose metabolism [23] but lately implicated as an indispensable element for NFkB activation and proinflammatory responses [21, 24]. Thus, GSK3β may serve as a potential target for treating podocyte injury and glomerular disease.

GSK3β is a druggable target with several clinically available drugs known to possess GSK3β inhibitory activities [25]. Among these, valproate, a short branched-chain fatty acid and an FDA approved first line anticonvulsant and mood stabilizer, is a potent inhibitor of GSK3β that can effectively block the activity of GSK3β at concentrations similar to those attained clinically [26, 27]. It has been known for many years that valproate confers a renoprotective effect on diverse experimental kidney diseases, including Adriamycin nephropathy [28] and diabetic nephropathy [29, 30], resulting in prominent improvement in kidney function, proteinuria and renal histology, including diminished renal inflammation and podocyte and glomerular injury. However, the underlying mechanism is not clear. It remains uncertain whether valproate confers a direct podocyte protective effect and whether its inhibitory activity on GSK3β is implicated. This study employed an *in vitro* model of podocyte injury elicited by lipopolysaccharide (LPS), a prototypical pathogen-associated molecular pattern found on the outer membrane of Gram-negative bacteria [31], to determine the direct effect of valproate on podocyte injury and to decipher the role of GSK3β signaling. The *in vitro* findings were validated in a murine model of LPS-elicited albuminuria and podocyte injury.

## RESULTS

### Valproate mitigates podocyte acquisition of de novo immune phenotypes upon LPS injury

**Figure 1:**
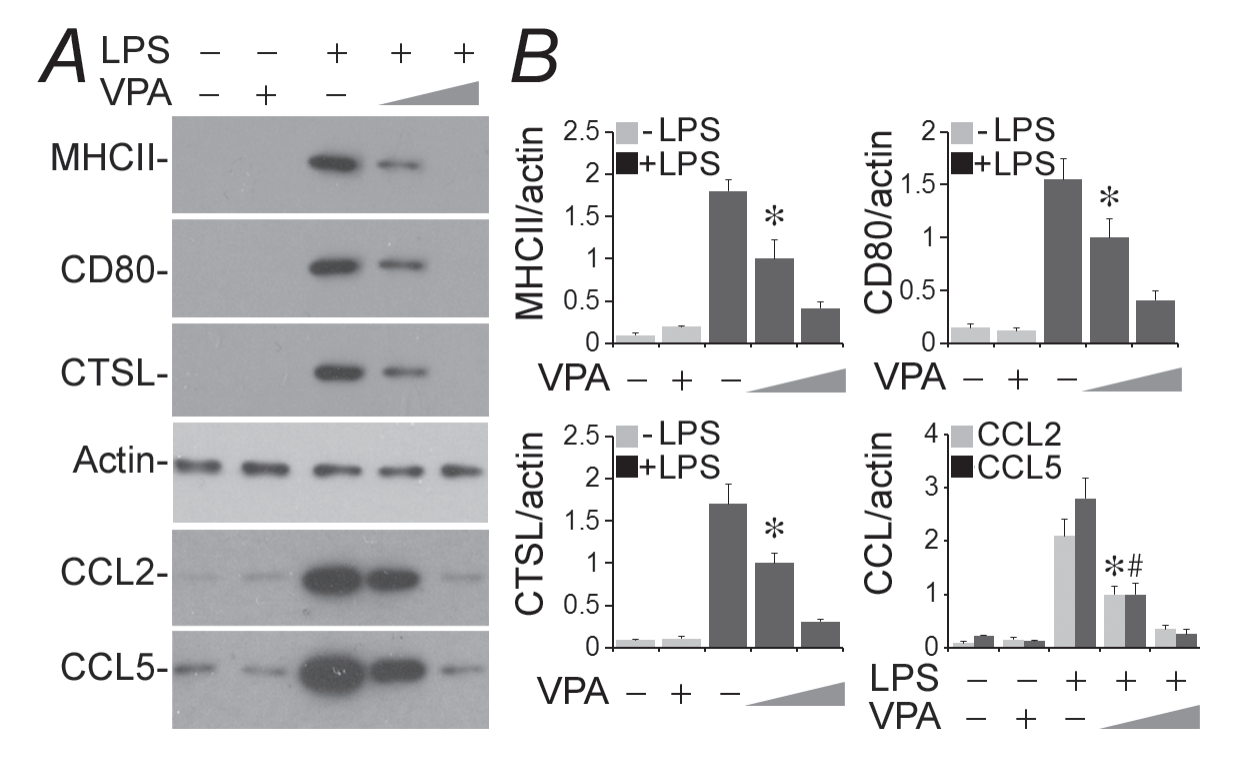
Valproate hinders podocyte acquisition of de novo immune phenotypes following LPS exposure Immortalized murine podocytes were cultured and differentiated under nonpermissive conditions. Cells were injured with LPS (100µg/ml) or vehicle for 24 h in the presence or absence of sodium valproate (VPA; 0.5 or 1.5 mM). **A.** Cell lysates or conditioned media were collected and prepared for immunoblotting for indicated proteins. **B.** Immunoblots were subjected to densitometric analysis and arbitrary units were expressed respectively as immunoblot densitometric ratios of diverse molecules to actin as folds of a designated group. *P < 0.05 versus the same molecule in other groups; ^#^*P* < 0.05 versus CCL5 levels in other groups (*n* = 3). CTSL, cathepsin L; LPS, lipopolysaccharide; VPA, sodium valproate.

Conditionally immortalized murine podocytes were cultured and differentiated under nonpermissive condition [32]. LPS is a typical injurious stimulus that is able to elicit podocyte injury and dysfunction both *in vivo* [33] and *in vitro* [3]. Indeed, following exposure to LPS (100µg/ml) for 24 h, podocytes newly expressed evident immune phenotypes that were either absent or scant under basal conditions. These include MHC II, costimulatory molecule CD80, lysosomal protease cathepsin L and proinflammatory cytokines like CCL2 and 5, as shown by immunoblot analysis of cell lysates or conditioned media (Figure 1). This inducible effect of LPS on podocyte expression of all immune molecules was substantially mitigated by concomitant treatment with valproate in a dose-dependent fashion as evidenced by immunoblot analysis (Figure 1A) followed by densitometry (Figure 1B).

### Valproate attenuates podocyte shape changes and actin cytoskeleton disorganization associated with LPS-induced acquisition of *de novo* immune phenotypes

**Figure 2:**
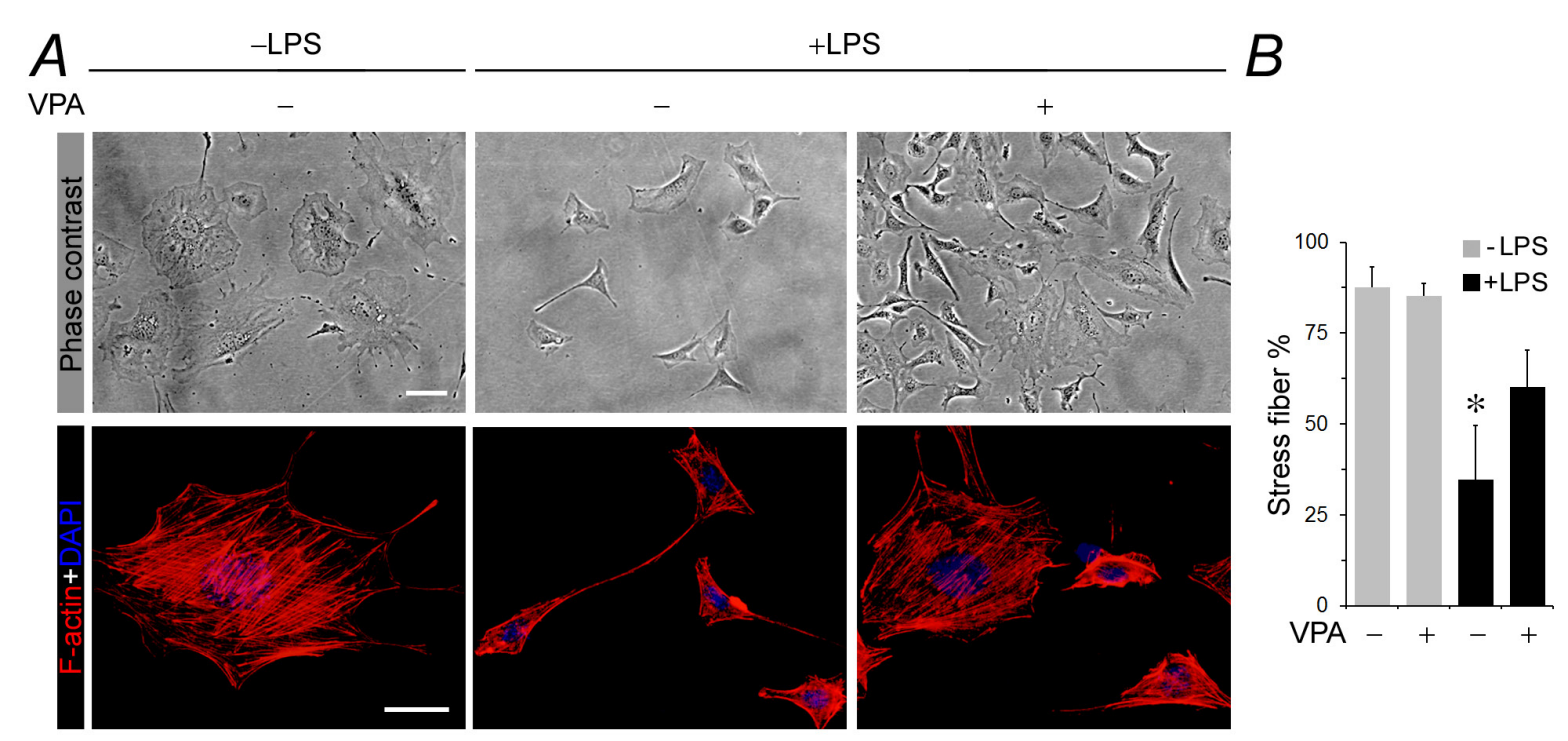
Valproate attenuates cellular shape changes, loss of stress fibers and disruption of actin cytoskeletal integrity in podocytes injured with LPS Immortalized murine podocytes were cultured and differentiated under nonpermissive conditions. Cells were injured with LPS (100µg/ml) or vehicle for 24 h in the presence or absence of sodium valproate (VPA; 1.5 mM). **A.** Represetative phase contrast micrographs show podocyte shape changes. Podocytes were large, flat and arborized cells with evidently rich stress fibers in cytoplasm and well-developed processes under basal condition. Following LPS exposure, podocytes demonstrated prominent cytologic changes, marked by podocyte shrinkage and a spindle-like or asterlike cell shape. Valproate co-treatment strikingly prevented aberrance in podocyte morphology. Bar = 10µm. Alternatively, podocytes were processed for fluorescent labeling of cytoskeletal filamentous actin (F-actin) with rhodamine conjugated phalloidin and counterstained with 4’,6-diamidino-2-phenylindole (DAPI). Representative fluorescent microscopic images show changes in F-actin cytoskeleton. Bar = 10µm. **B.** The percentage of stress fibers under fluorescent microscopy were analyzed and estimated. *P < 0.05 *versus* other groups (*n* = 4). DAPI, 4’,6-diamidino-2-phenylindole; LPS, lipopolysaccharide; VPA, sodium valproate.

Evidence suggests that podocyte acquisition of *de novo* immune phenotypes is associated with cytoskeletal disarrangement and podocyte dysfunction [11, 12, 18]. As a matter of fact, a number of immune molecules, like MHC II [34], CD80 [10, 11] and cathepsin L [12, 18], function at the interface between innate/adaptive immunity and cytoskeletal remodeling and are known to cause podocyte cytoskeleton disorganization. Shown in Figure 2A, under basal conditions, podocytes exhibited as large, flat and arborized cells with abundant stress fibers in cytoplasm and well-developed processes, as demonstrated by phase contrast microscopy (Figure 2A). Labeling of filamentous actin (F-actin) with rhodamine-conjugated phalloidin revealed stretched cellular shape and intense phalloidin-labeled ventral stress fibers with long paralleled cortical stress fibers in normal podocytes (Figure 2A). The percentage of F-actin under fluorescent microscopy were analyzed (Figure 2B). Following LPS exposure, podocytes demonstrated prominent morphologic changes, marked by podocyte shrinkage and a spindle-like or asterlike cell shape, in parallel with drastic disruption of actin cytoskeleton that manifested as increased expression of cortical filaments, diminished ventral stress fibers, more transverse arcs, and sporadic short dorsal stress fibers (Figure 2A and 2B). This was concurrent with *de novo* expression of MHC II, CD80 and cathepsin L as described above. Valproate co-treatment strikingly prevented stress fiber disruption and aberrance in podocyte morphology (Figure 2A and 2B).

### Valproate counteracts GSK3β overactivity and NFkB phosphorylation in LPS-injured podocytes

**Figure 3:**
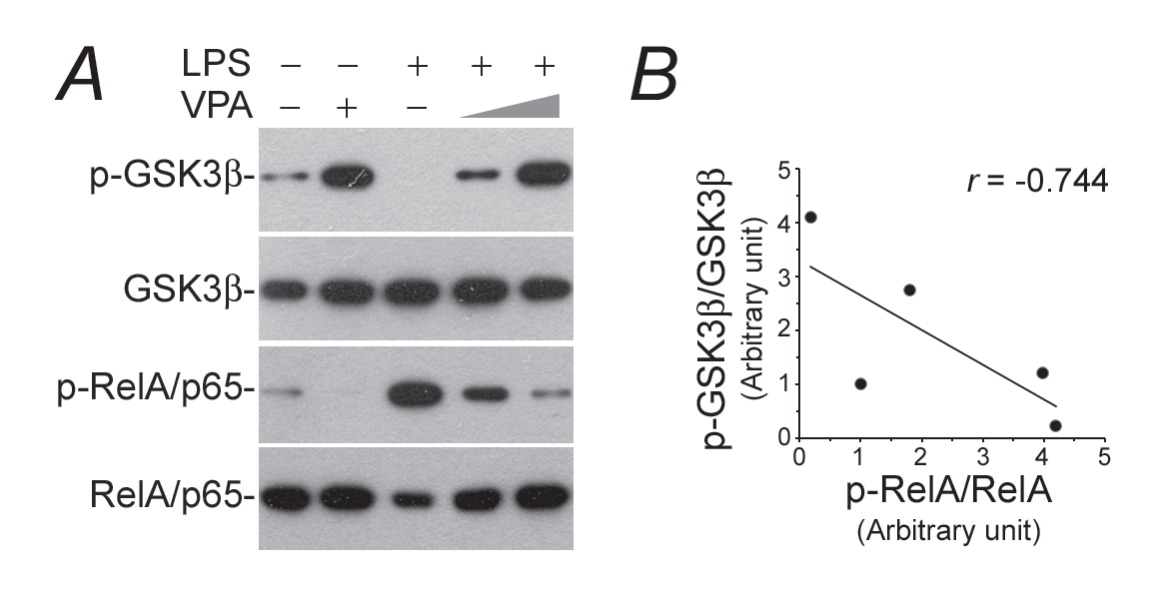
The LPS-elicited GSK3β overactivity and NFkB hyperactivation in podocytes is obliterated by valproate Podocytes were treated as elaborated in Figure 1. **A.** Cell lysates were collected and prepared for immunoblotting for phosphorylated GSK3β at serine 9 residue, phosphorylated NFkB RelA/p65 at serine 467 residue and other indicated proteins. **B.** Immunoblots were subjected to densitometric analysis and arbitrary units of p-GSK3β and p-RelA were expressed respectively as immunoblot densitometric ratios of p-GSK3β/GSK3β and p-RelA/p65/RelA/p65 as folds of the control group. Linear regression analysis indicated a statistically significant (*P* < 0.05) inverse correlations between p-GSK3β and p-RelA/p65 in podocytes. LPS, lipopolysaccharide; VPA, sodium valproate.

NFkB activation is a prerequisite for *de novo* expression of diverse immune molecules, whose transcription are all NFkB-dependent [20]. To discern if the valproate-abrogated podocyte *de novo* expression of immune phenotypes stems from a possible effect on NFkB activation, cell lysates were subjected to immunoblot analysis for phosphorylation of NFkB RelA/p65 at serine 467 residue, which plays a key role on specifying the expression of proinflammatory molecules involved in kidney injury and podocyte dysfunction [22, 35]. Shown in Figure 3A, LPS triggered considerable RelA/p65 phosphorylation and this effect was hindered by valproate in a dose dependent manner, accompanied with inhibition of GSK3β, a molecular target of valproate [26], as indicated by increased phosphorylation at the serine 9 inhibitory site. Previous data suggest that GSK3β is an indispensable element for NFkB activation [21]. In agreement, linear regression analysis illustrated an inverse correlation between p-GSK3β and p-RelA/p65 in podocytes (Figure 3B), namely a positive correlation between GSK3β overactivity and NFkB RelA/p65 activation.

### Ectopic expression of the constitutively active GSK3β blunts the podocyte protective activity of valproate

**Figure 4:**
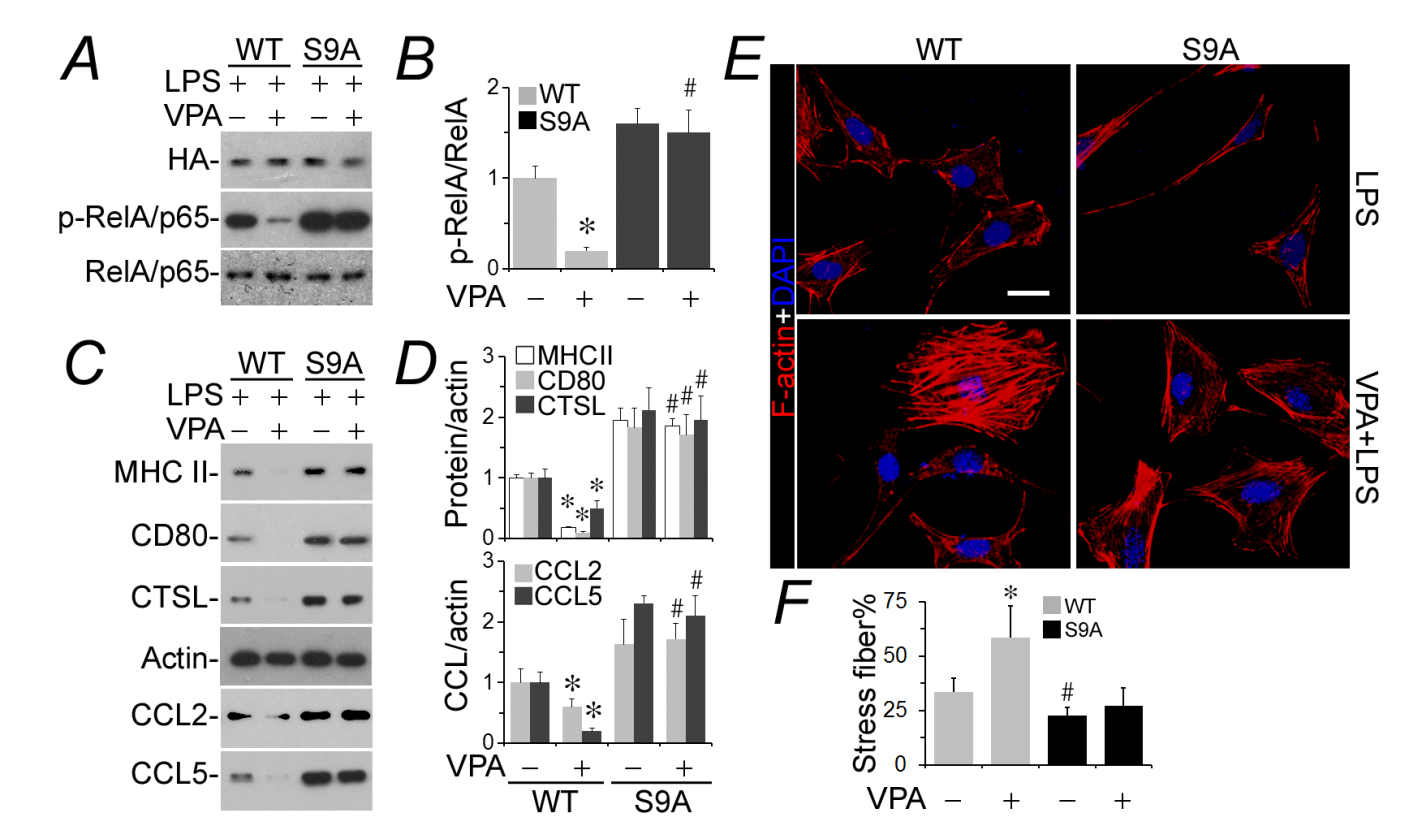
The podocyte protective activity of valproate is blunted in podocytes expressing a constitutively active mutant of GSK3β Differentiated murine podocytes were subjected to liposome-mediated transient transfection with vectors encoding the HA-conjugated wild-type GSK3β (WT) or mutant GSK3β (S9A), in which the regulatory serine 9 residue is replaced by alanine so that GSK3β becomes uninhibitable. Cells were treated with LPS (100 µg/ml) for 24 h after transfection in the presence or absence of sodium valproate (VPA; 1.5 mM). **A.** Cell lysates were collected and prepared for immunoblotting for phosphorylated NFkB RelA/p65 at serine 467 residue or indicated molecules. **B.** Immunoblots were subjected to densitometric analysis and arbitrary units were expressed respectively as immunoblot densitometric ratios of p-NFkB RelA/p65 to NFkB RelA/p65 as folds of the control group. *P < 0.05 *versus* the level of the same molecule in the non-VPA treated WT group; ^#^Not significant *versus* the level of the same molecule in the non-VPA treated S9A group (*n* = 3). **C.** Cell lysates or conditioned media were collected and prepared for immunoblotting for indicated immune molecules. **D.** Immunoblots were subjected to densitometric analysis and arbitrary units were expressed respectively as immunoblot densitometric ratios of diverse molecules to actin as folds of the control group. *P < 0.05 *versus* the level of the same molecule in the non-VPA treated WT group; ^#^Not significant *versus* the level of the same molecule in the non-VPA-treated S9A group (*n* = 3). **E.** Cells were fixed and processed for fluorescent labeling of F-actin and counterstained with 4’,6-diamidino-2-phenylindole (DAPI). Represetative fluorescent microscopy shows changes in F-actin cytoskeleton. Bar = 10µm. **F.** The percentage of stress fibers under fluorescent microscopy were analyzed and estimated. *P < 0.05 *versus* non-VPA-treated WT cells; ^#^*P* < 0.05 versus non-VPA-treated WT cells; ^#^Not significant *versus* VPA-treated S9A cells (*n* = 4). CTSL, cathepsin L; DAPI, 4’,6-diamidino-2-phenylindole; LPS, lipopolysaccharide; HA, hemagglutinin; VPA, sodium valproate.

To substantiate if inhibitory phosphorylation of GSK3β at serine 9 plays a key role in transmitting the podoprotective activity of valproate, podocytes were subjected to transient transfection of plasmids encoding a hemagglutinin-conjugated wild-type GSK3β (WT) or mutant GSK3β (S9A), in which the regulatory serine 9 residue is replaced by alanine so that GSK3β becomes uninhabitable and constitutively active. Cells were then injured with LPS in the presence or absence of valproate. Shown by immunobot analysis, the LPS-induced NFkB activation (Figure 4A and 4B) and *de novo* expression of all immune phenotypes (Figure 4C and 4D) were apparently enhanced in podocytes expressing S9A as compared with those expressing WT, denoting that GSK3β overactivity amplifies the podocyte response to LPS. In accordance with the augmented NFkB activation and expression of all immune molecules in podocytes expressing S9A, the LPS-elicited actin cytoskeleton disorganization was sensitized in S9A-expressing cells as compared with that in WT-expressing cells (Figure 4E). This effect was quantified by analyses of the percentage of F-actin under fluorescent microscopy (Figure 4F). Valproate drastically hindered NFkB activation (Figure 4A and 4B) and podocyte *de novo* expression of immune molecules (Figure 4C and 4D) in WT-expressing following LPS exposure and mitigated the LPS impaired actin cytoskeleton integrity (Figure 4E and 4F). In contrast, the effect of valproate was largely blunted in S9A-expressing cells.

### Valproate therapy mitigates podocyte acquisition of immune phenotypes and attenuates albuminuria in LPS-injured mice

**Figure 5:**
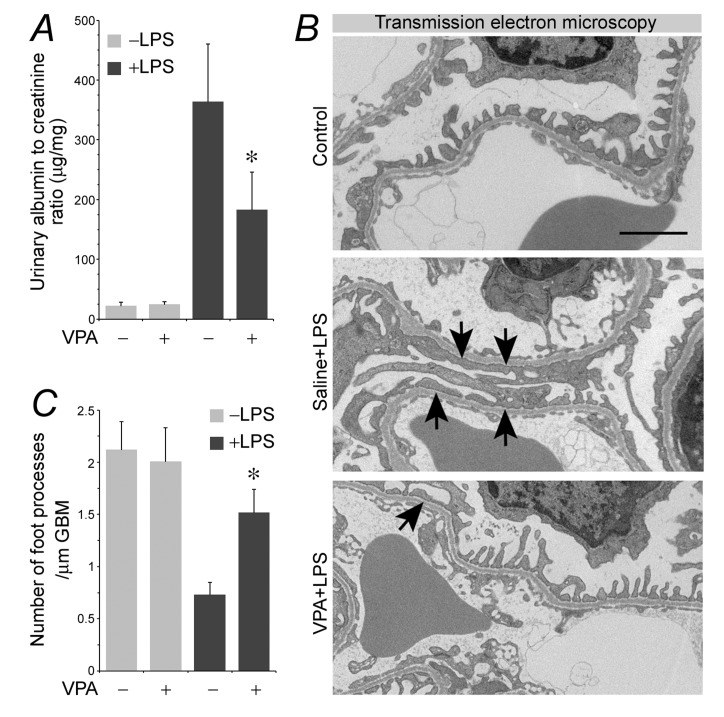
Valproate protects against podocyte injury and albuminuria in LPS-injured mice Mice were treated with sodium valproate (200mg/kg) or saline (100µl) followed by LPS (200µg) injury or vehicle treatment. Mice were followed for 24 h. **A.** Urine was collected and processed for urine albumin ELISA assay followed by adjustment for urine creatinine concentrations. *P < 0.05 *versus* non-VPA-treated and LPS-injured mice (*n* = 6). **B.** Transmission electron microscopy of kidney glomeruli demonstrated marked foot process effacement (black arrows) in LPS-injured mice. This effect was prevented by valproate treatment. **C.** Morphometric analysis of the number of foot processes per µm glomerular basement membrane (GBM) revealed by electron microscopy (right panel). Bar = 2 µm.*P < 0.05 versus other groups (*n* = 6). LPS, lipopolysaccharide; VPA, sodium valproate.

**Figure 6:**
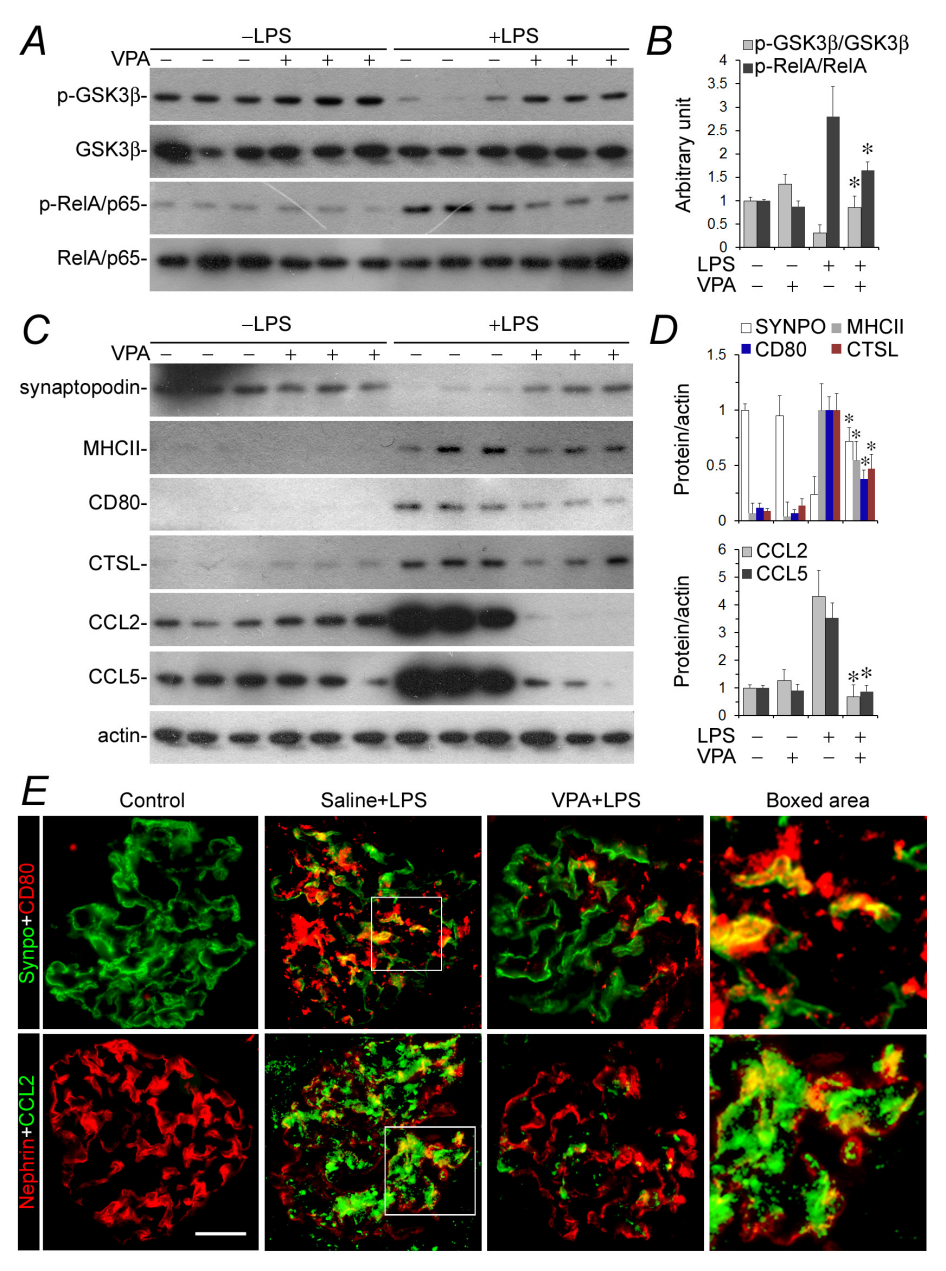
GSK3β overactivity and NFkB activation in glomeruli in LPS-injured mice were mitigated after valproate therapy, resulting in a blunted podocyte acquisition of immune phenotypes and podocytopathy Mice were treated as stated in Figure 5 and mouse kidneys were procured for further examination. **A.** and **C.** Isolated glomeruli were processed for immunoblot analysis for diverse molecules. **B.** and **D.** Immunoblots were subjected to densitometric analysis and arbitrary units were expressed respectively as immunoblot densitometric ratios of diverse molecules to actin as folds of the control group. *P < 0.05 *versus* the level of the same molecule in LPS-injured mice without VPA treatment (*n* = 6). **E.** Frozen sections of mouse kidneys were processed for dual color fluorescent immunohistochemistry staining for indicated immune molecules and podocyte marker proteins. Representative microscopic images were shown. Bar = 50 µm. CTSL, cathepsin L; LPS, lipopolysaccharide; SYNPO, synaptopodin; VPA, sodium valproate.

To test if valproate is able to protect against podocyte injury *in vivo*, the murine model of LPS-elicited podocytopathy was employed. LPS has been reproducibly demonstrated by previous studies to cause acute podocyte injury and albuminuria in mice [11, 36, 37]. Indeed, 24 h after LPS injury, albuminuria was evident in our mice (Figure 5A), in parallel with signs of podocyte injury, marked by podocyte foot process effacement under transmission electron microscopy (Figure 5B and 5C). This was associated with GSK3β overactivity in glomeruli, reflected by reduced inhibitory phosphorylation of GSK3β as probed by immunoblot analysis of isolated glomeruli (Figure 6A and 6B). Consistent with *in vitro* findings, GSK3β overactivity in glomeruli resulted in NFkB RelA/p65 phosphorylation and activation (Figure 6A and 6B) as well as an induced glomerular expression of diverse podocytopathic immune molecules, including MHCII, CD80, cathepsin L and proinflammatory cytokines CCL2 and 5 (Figure 6C and 6D), as shown by immunoblot analysis of isolated glomeruli followed by densitometry. This was paralleled by prominent podocyte injury, marked by loss of podocyte marker proteins like synaptopodin (Figure 6C and 6D). Dual-color immunohistochemistry staining indicated that immune molecules, like CD80 and CCL2, were evidently detected in glomerular cells positive for podocyte-specific markers, such as synaptopodin and nephrin, thus indicative of a pattern of podocyte distribution (Figure 6E). Valproate treatment substantially attenuated albuminuria (Figure 5A) and prevented podocyte injury with significant improvement in podocyte injury and foot process effacement (Figure 5B and 5C). Mechanistically, valproate therapy diminished GSK3β overactivity in renal glomeruli in mice following LPS injury, mitigated NFkB activation (Figure 6A and 6B), and offset the LPS-elicited expression of diverse immune molecules in glomeruli and podocytes (Figure 6C∼6E).

## DISCUSSION

Valproate, an FDA approved drug, has been used for almost 50 years as the first line anti-convulsant for the treatment of epilepsy and also as a mood-stabilizer to control bipolar affective disorder [38], [39]. Exposure to valproate can trigger a multitude of cellular responses that may be responsible for its clinical efficacy and multiple actions in diverse organ systems [38]. Burgeoning evidence recently supports a protective activity of valproate in diverse somatic diseases, including kidney disease [28-30]. Although a number of cell signaling transducers, including inositol, histone deacetylase (HDAC), GSK3β and others, have been implicated as molecular targets of valproate [38], the underlying mechanisms responsible for the beneficial effect of valproate in diverse experimental diseases are largely unknown. The present study for the first time provided evidence in support of a direct podocyte protective effect of valproate that is likely mediated *via* intercepting the GSK3β facilitated NFkB activation and the ensuing NFkB-dependent *de novo* acquisition of immune phenotypes.

Our finding that valproate possesses a podocyte protective activity both *in vitro* in cultured podocytes and *in vivo* in murine models of LPS podocytopathy is congruent to several recent pre-clinical observations suggesting that valproate exerts a glomerular protective and proteinuria reducing effect [28-30]. Indeed, in rats with streptozotocin-elicited diabetes [29], oral treatment with low or high dose of valproic acid attenuated physiologic signs of diabetic nephropathy, including kidney dysfunction and albuminuria, and improved histologic evidence of glomerular injury, such as glomerular matrix accumulation and enlargement, glomerular cell apoptosis, and ultrastructural lesions in podocytes, marked by podocyte foot process effacement. In agreement, in another study on murine models of Adriamycin nephropathy [28], both preventive and rescue treatment with valproic acid were able to drastically attenuate urinary excretion of albumin and ameliorate renal histologic injuries, including glomerulosclerosis, renal inflammation, fibrosis and podocyte detachment and foot process effacement. Although both studies demonstrated an appealing renoprotective property of valproate, the underlying mechanism is unknown. Of note, podocyte injury and proteinuria could be a bystander phenomenon caused by systemic factors ensuing metabolic, hematopoietic or immune dysregulations [40]. Correction of diabetes or systemic hyperglycemia *per se* is known to improve and even reverse glomerular damage and podocyte injury [41]. Likewise, adoptive transfer of myeloid-derived suppressor cells has been shown to be sufficient to attenuate podocyte injury, glomerulopathy and proteinuria in experimental glomerulopathies [42]. At least, there is evidence suggesting an anti-diabetic effect of valproate that is able to correct hyperglycemia in diabetic models [43]. Moreover, valproate is known to have a potent immunoregulatory activity that can lead to profound alternations in immune response in both animal models and in humans [44]. Given these confounding effects of valproate on immune system or on diabetes, it remains to be clarified if the anti-protenuric and glomerular protective activity of valproate is attributable to an effect on podocyte autonomous injury. Our present study took advantage of the *in vitro* and *in vivo* models of the LPS-inflicted podocytopathy in the absence of any systemic cues, like hyperglycemia or immune factors, and unequivocally demonstrated a protective effect of valproate on podocyte autonomous injury.

How valproate protects against podocyte injury has been barely examined before. Valproate is a well-established quintessential inhibitor of GSK3β [26, 27], a kinase that has been implicated in podocyte autonomous injury via integrating multiple podocytopathic cellular pathways [45], including proinflammatory NFkB activation and the ensuing *de novo* expression of NFkB-dependent immune molecules in podocytes [22, 45]. The mode of action by which valproate blocks GSK3β activity is still a matter of debate, but predominant evidence points to an induced inhibitory phosphorylation of GSK3β at the serine 9 residue [27], a mechanism that is also adopted by lithium, another well characterized GSK3β inhibitor. In support of this, in valproic acid-associated fetal cardiac teratogenicity, the severity of the defect is correlated to the phosphorylation state of GSK3β serine 9 [46]. Moreover, in neuroblastoma cells, valproate treatment activated Akt and subsequently augmented inhibitory phosphorylation of GSK3β [27]. In line with these reports, our present study found that the beneficial activity of valproate was offset in podocytes ectopically expressing a mutant GSK3β, in which the regulatory serine 9 residue is changed to alanine so that GSK3β became uninhibitable and constitutively active, entailing that inhibitory phosphorylation is likely essential for the protective effect of valproate. *In vivo*, in LPS-injured mice, valproate treatment prevented albuminuria and this effect was associated with GSK3β inhibition. LPS is also able to induce acute kidney injury (AKI). Inhibition of GSK3β has been shown to confer a renoprotective action in various models of AKI. Provided the inhibitory effect of valproate on GSK3β, it is highly possible that valproate treatment may also be able to improve kidney dysfunction and AKI in the LPS-injured mice. This merits further studies. In addition to GSK3β, valproate may also target other cell signaling molecules in podocytes. Admittedly, accumulating data pointed to valproate as an inhibitor of HDACs [47]. In consistency, the aforementioned two studies on the effect of valproic acid in experimental diabetic [29] or Adriamycin [28] nephropathy also demonstrated association between kidney protection and increased histone acetylation in kidney cells and thus assumed that inhibition of HDAC might mediate glomerular protection by valproic acid. Such conclusions are, nevertheless, arbitrary and inconclusive because no causal relationship was established in both studies [28, 29]. Latest research work did uncover that a couple of members of HDAC family may play a direct pathogenic role in podocyte injury [48, 49]. These include HDAC4 [48] and HDAC9 [49], which belong to the class II HDACs family. However, valproate, at clinically achievable concentrations, is a typical inhibitor of the class I HDACs [50, 51]. In order to suppress the activity of class II HDACs, valproate requires more than 20 times the clinical concentrations [51]. Thus, it remains doubtful if HDAC inhibition mediates the direct podocyte protective effect of valproate. Our study was not designed and thus unable to determine whether inhibition of HDACs also contributes, at least in part to, the podoprotective effect of valproate. But in S9A-expressing podocytes, the protective efficacy of valproate was abolished. This finding apparently implies that GSK3β is likely the key, if not the sole, target of valproate that is principally responsible for valproate’s podocytoprotective activity. Even if HDAC inhibition is implicated in the podocyte protective effect of valproate, it might be distal or downstream to GSK3β inhibition, as there is evidence suggesting that multiple HDACs [52-54], including HDAC4 [53] are direct targets of GSK3β and their activities and expression levels are under the control of GSK3β.

In summary, our study for the first time indicates that valproate directly protects against podocyte injury and hampers podocyte acquisition of *de novo* immune phenotypes *via* intercepting GSK3β-facilitated NFkB activation. In murine models of LPS podocytopathy, valproate therapy ameliorates podocyte injury and albuminuria. Our study may pave the way to develop a novel, feasible and affordable therapeutic modality based on valproate for the treatment of proteinuric glomerulopathies.

## MATERIALS AND METHODS

### Cell culture

Conditionally immortalized mouse podocytes in culture (courtesy of Dr. Stuart Shankland, University of Washington, Seattle, WA) [55] between passages 21 and 25 were used. Podocytes were cultured in RPMI 1640 medium (Invitrogen) supplemented with 10% FBS in a humidified incubator with 5% CO2. The cells were cultured at 33 °C with 50 units/ml recombinant mouse interferon-g (Millipore,Billerica, MA) on collagen-coated plastic Petri dishes and were transferred to a 37 °C incubator without interferon-g to induce differentiation for 14 days. Podocytes were pretreated with valproic acid (sodium salt, Sigma, St. Louis, USA; 0.5 or 1.5 mM) or vehicle for 30 min and then stimulated with 100 µg/ml LPS (serotype: E. coli 0111: B4, Sigma) or saline for 24 h.

### Transient transfection

The eukaryotic expression vectors encoding the hemagglutinin (HA) conjugated constitutively active (S9A) GSK3β mutant (S9A-GSK3β-HA/pcDNA3) or wild-type (WT) GSK3β (WT-GSK3β-HA/pcDNA3) were provided by Dr. Johnson (Birmingham, AL) [55]. Transient transfection was carried out by using Lipofectamine 2000 (Invitrogen, Carlsbad, CA, USA) as described previously [55]. In brief, podocytes were cultured under permissive conditions at 50∼70% confluence in the absence of antibiotics. The plasmid-Lipofectamine 2000 complexes were prepared and applied to proliferating podocytes for transfection. The ratio of Lipofectamine 2000 to vectors was optimized by a series of pilot experiments for each study until the best transfection efficiency or the best gene-silencing efficiency was achieved. After transfection, the cells were cultured under nonpermissive conditions in normal growth medium for 48 h before transfection efficiency was assessed by immunocytochemistry staining or immunoblot analysis for target molecules. Cells were then subjected to LPS, vehicle or other treatments.

### Animal studies

Animal studies were approved by the institutional Animal Care and Use Committees, and they conform to the US Department of Agriculture regulations and the NIH’s Guide for human care and use of Laboratory Animals. Male FVB mice aged 8 weeks were randomized to each of the following treatments. Sodium valproate (200mg/kg) or saline (100µl) was given *via* intraperitoneal injection based on previous reports [28] 1 h before intraperitoneal injection of saline (100µl) or LPS (200µg; serotype: E. coli 0111:B4, Sigma) [36]. Mice were followed for 24 h before they were killed and the kidneys resected for further investigation. Urine was collected 24 h after LPS injection. Six mice were randomly assigned to each group.

### Urine analyses

Urine albumin levels were measured using mouse albumin ELISA quantitation kit (Bethyl Laboratories, Montgomery, TX, USA). Urine creatinine was determined by a creatinine assay kit (BioAssay Systems, Hayward, CA, USA). To rule out the influence of glomerular filtration rate on urinary excretion of albumin, urine albumin levels were adjusted by urine creatinine levels and presented as urine albumin-to-creatinine ratios.

### Transmission electron microscopy

For electronmicroscopy, kidney cortical tissues were cut into small pieces (1 mm3), fixed with 2.5% glutaraldehyde, and embedded in Epon 812 (Polysciences Inc.,Warrington, PA). Transmission electron micrographs were obtained using an EM-10 microscope (Zeiss) operated at 60 kV. The number of foot processes per 10 μm of glomerular basement membrane (GBM) in different electron microscopic fields was measured. The result was divided by the total length of the GBM and expressed as the number per micrometer of GBM length as previously described [55].

### Immunofluorescent staining

Cultured cells or frozen kidney cryostat sections were fixed and processed for fluorescent staining. For dual color fluorescent immunohistochemistry staining, samples were stained with primary antibodies and followed by applying the Alexa Fluor-conjugated secondary antibodies (Invitrogen). As a negative control, the primary antibodies were replaced by preimmune IgG from the same species; no staining occurred. Finally, samples were counterstained with 4’,6-diamidino-2-phenylindole (DAPI) and mounted with Vectashield mounting medium (Vector Laboratories, Burlingame, CA, USA). For fluorescence microscopy, all sections were analyzed at the same time to exclude artifacts due to variable decay of the fluorochrome. Sections were examined using an Olympus fluorescence microscope equipped with a Spot II digital camera. For dual-color staining, images were acquired sequentially to avoid dye interference. ImageJ software was used for post processing of the images, e.g. scaling, merging, and colocalization analysis.

### Fluorescent labeling of cytoskeletal filamentous actin in podocytes

Following various treatments, podocytes in culture were fixed in 4% paraformaldehyde in PBS and permeabilized filamentous actin (F-actin) was stained by incubation at 4°C with rhodamine conjugated phalloidin (Cytoskeleton Inc., Denver, CO). Then, cells were counterstained with 4’,6-diamidino-2-phenylindole (DAPI) and mounted with Vectashield mounting medium. Images were documented using an Olympus fluorescence microscope equipped with a Spot II digital camera. The percentage of F-actin or stress fibers under fluorescent microscopy were analyzed and quantitated as described elsewhere [56].

### Glomerular isolation

Glomerular isolation was conducted as reported elsewhere [22]. In brief, mice were anesthetized and kidney perfused with 5ml of phosphate-buffered saline containing 8×10^7^ Dynabeads M-450 (Dynal Biotech ASA, Oslo, Norway). After perfusion, the kidneys were resected and kidney cortices minced into 1-mm^3^ pieces and digested in collagenase A (1 mg/ml, Sigma) at 37^°^C for 30 min with gentle shaking. The tissue was pressed gently through a 100 μm cell strainer (BD Falcon, Bedford, MA, USA) and glomeruli containing Dynabeads were then gathered using a magnetic particle concentrator. An aliquot (1:1500) of the glomerular isolate was visualized under a microscope to ensure that the sample contained fewer than five tubular fragments per x200 field. Most isolated glomeruli (80%) were decapsulated, which was similar to what had been reported previously [55].

### Western immunoblot

Cultured cells or kidney tissues were lysed in Radioimmunoprecipitation assay (RIPA) buffer supplemented with protease inhibitors. Cell lysates and conditioned media were subjected to immunoblot analysis as previously described [35]. The antibodies against MHC II, cathepasin L, and actin were purchased from Santa Cruz Biotechnology (Santa Cruz, CA, USA), those against CD80, CCL2 and CCL5 from R&D Systems (Minneapolis, MN, USA), and that against p-GSK3β, GSK3, p-RelA/p65 and RelA/p65 from Cell Signaling Technology (Danvers, MA, USA).

### Statistical analyses

For immunoblot analysis, bands were scanned and the integrated pixel density was determined using a densitometer and the ImageJ analysis program. All data are expressed as mean ± SD. Unless otherwise indicated, all experimental observations were repeated three times. Statistical analysis of the data from multiple groups was performed by repeated measures ANOVA followed by Fisher’s Least Significant Difference (LSD) tests. Data from two groups were compared by Student’s t-test. Linear regression analysis was applied to examine possible relationships between two parameters. *P* < 0.05 was considered significant.
